# Stereotactic radiosurgery for multiple small brain metastases using gamma knife versus single‐isocenter VMAT: Normal brain dose based on lesion number and size

**DOI:** 10.1002/acm2.70065

**Published:** 2025-03-19

**Authors:** Abram Abdou, Timoteo Almeida, Elizabeth Bossart, Irene Monterroso, Eric A. Mellon

**Affiliations:** ^1^ Department of Radiation Oncology Sylvester Comprehensive Cancer Center University of Miami Miller School of Medicine Miami Florida USA

**Keywords:** dosimetry, gamma knife, linear accelerator, radiosurgery, SRS

## Abstract

**Purpose:**

The study evaluates rapid linear accelerator (Linac) single isocenter stereotactic radiosurgery (SRS) with Hyperarc for large target numbers. We compared to Gamma Knife (GK), which suffers from long treatment times and investigated causes of differences.

**Methods:**

Linac SRS and GK treatment plans for patients receiving 18 Gy to the gross tumor volume (GTV) were evaluated for mean brain dose and volume of brain receiving 12 Gy or more (V12 Gy) as toxicity correlates. Further investigations included patient‐based and simulations of 1–33 brain metastases to compare the ability of Linac SRS and GK to separate adjacent and distant lesions.

**Results:**

For three patients (33, 33, and 18 metastases), GK reduced mean brain dose (2.89 Gy, 2.38 Gy, 2.79 Gy) compared to 2.5 mm microMLCs (4.36 Gy, 4.75 Gy, 4.26 Gy, *p* = 0.027) and 5 mm MLCs (4.71 Gy, 5.22 Gy, 4.60 Gy, *p* = 0.024). GK also improved V12 Gy (13.29 cc, 11.62 cc, 33.79 cc) compared to microMLC (25.31 cc, 30.91 cc, 54.71 cc, *p* = 0.019) and MLC (31.69 cc, 33.68 cc, 54.71 cc). This must be balanced with GK treatment times (5–11 h). GK achieved 50% prescription line separation at smaller distances (1.8–7.6 mm) than microMLC (7.7–10.2 mm) or MLC (8.8–12.2 mm) for 0.5–1.0 cm targets (4–8 mm collimator single shot). For 1.5 cm targets (16 mm shot) results were mixed (GK 5.4–17 mm, microMLC 9.5‐11.2 mm, MLC 9.5–11.3 mm). A 7.7 cm simulation cube was then incrementally filled with 0.5 cm or 1.0 cm equidistant targets. GK plan mean brain dose increased 0.04 Gy/target (1.08 Gy mean/27 targets) compared to 0.14 Gy/target for microMLC (3.78 Gy mean/27 targets) for 0.5 cm targets, with differences diminishing for 1.0 cm targets (GK 0.15 Gy/target, microMLC 0.17 Gy/target).

**Conclusions:**

For numerous small metastases GK improves dosimetry but has exceedingly long treatment times. GK improves dose separation for adjacent lesions < 1.0 cm and conformity for small (∼0.5 cm) targets. GK and Linac differences are small for individual targets but compound over many targets. V12 Gy limits in the NCIC CE.7 trial protocol mandate dose modifications for Linac SRS but not GK.

## 1 INTRODUCTION

As systemic cancer therapies improve and overall survival increases, it is more common to encounter patients with multiple brain metastases (BM) and good performance status.^1^, ^2^ For such patients, stereotactic radiosurgery (SRS) is increasingly utilized in the hope of preserving neurocognitive function while locally controlling the disease.^3^, ^4^ Another motivation is that following whole brain radiation, metastases have the potential to recur, and outcomes are suboptimal when considering a second round of whole‐brain radiation.^5^, ^6^ This leads to increased utilization of SRS as both primary and salvage options for BM.^7^, ^8^, ^9^ Several radiosurgery platforms can be considered for this purpose.^10^

One commonly employed option is Gamma Knife (GK, Elekta, Stockholm, Sweden).^11^, ^12^ GK is a longstanding option for high‐quality SRS, although it usually implies rigid fixation and increasing treatment times per lesion.^13^ Recent linac radiosurgery systems have combined mask‐based immobilization with the capability to treat a possibly unlimited number of targets simultaneously with multi‐leaf‐collimator (MLC) in a single isocenter plan as quickly as ∼15 min.^14^ Questions remain about the ability of single isocenter linac to deliver the same radiation plan quality as GK or other multi‐isocentric setups. Metrics such as mean brain dose and volume of brain receiving 12 Gy or more (V12 Gy) are commonly used to assess plan quality, as they are linked to neurocognitive toxicity and radiation necrosis risk.^15^, ^16^

When using single isocenter MLC linac systems, there is transmission of dose through the MLC or leakage between individual leaves that may increase whole brain radiation dose.^17^ While a few manuscripts have determined that single isocenter MLC linac systems can treat increasing numbers of brain metastases, it is unclear if there is a target number or volume limit where the MLC‐based plans are no longer of sufficient quality.^18^, ^19^ This study was motivated by three recent patients with 18–33 brain metastases who had prior whole brain radiation and good performance status where we had to consider either extremely long GK delivery, or an uncertain ability of single isocenter linac radiosurgery to provide quality treatment to many targets, a second course of whole brain radiation therapy, or supportive care alone. The primary goal of this study is to evaluate and compare the dosimetric outcomes of single‐isocenter linac radiosurgery and GK for patients with a high number of brain metastases, while identifying key factors contributing to the differences in plan quality between these treatment modalities. This study uniquely emphasizes how lesion characteristics such as orientation, size, and number, as well as treatment planning approaches impact mean brain dose and V12 Gy, metrics linked to neurocognitive outcomes and radiation toxicity. This targeted approach provides insights into the dosimetric trade‐offs and clinical considerations involving treatment modality.

As such, we compared treatment plans for the HyperArc (Varian, Palo Alto, CA) single isocenter linac system on both a TrueBeam platform and an Edge platform, and GK. To evaluate the causes of plan quality differences between the two systems we performed additional simulations. To isolate the effects of MLC size on plan quality, plans were generated with both 2.5 mm microMLC (Edge) and standard 5 mm MLC (TrueBeam) leaf widths. Then, we generated plans for these patients adding in the metastases one‐by‐one to identify potential numerical target limits for Linac SRS to deliver sufficient plan quality simultaneously. Next, to investigate the ability of GK and MLCs to separate doses between two adjacent lesions, we simulated two adjacent lesions of different sizes at different distances and orientations and measured the distance required to separate the 50% prescription isodose line. Finally, we generated a homogeneous brain sized grid of simulated brain metastases, each well separated and equally spaced, to systematically measure the effects of lesion size and ability of MLCs to separate many targets on plan metrics.

## 2 METHODS

All plans were generated in Leksell GammaPlan (v11.3.2) for GK Perfexion or Eclipse (v16.1.1) for Linac SRS. MicroMLC plans were generated using the Varian HD120 MLC (2.5 mm center MLC width) and MLC plans were generated using the Varian Millennium 120 MLC (5.0 mm center MLC width).^20^ All treatments for GK were planned with a Cobalt source that had a dose rate of 2.343 Gy/min. Plans were then exported for comparison to MIM (MIM Software Inc., Beachwood, OH, USA). GK beam‐on times were recorded for each plan.

### 2.1 Patient plans

We identified and selected cases treated with single fraction radiosurgery of 18 Gy to gross tumor volume (GTV). Patient 1 had 33 BM with cumulative GTV volume 3.9 cc. Patient 2 had 33 BM with cumulative GTV volume 3.31 cc. Patient 3 had 18 BM with a larger cumulative GTV volume of 5.64 cc. Histograms showing lesion size distribution has been added as supplementary file (Figure S1,S2,S3). These cases were acquired from a database of BM patients, ensuring all personal identifiers were removed to maintain patient confidentiality. Institutional Review Board permission was granted under a retrospective protocol.

All plans were generated by the same individual. Linac SRS plans were optimized for 100% of the dose to cover a minimum of 99.5% of the target using Eclipse's HyperArc Volumetric Modulated Arc Therapy (VMAT) automated planning feature and using the AcurosXB V16.1 calculation algorithm. GK plans were generated manually using mixed shots and isodose prescription 50–80% to provide high conformity with 99.5%–100% target coverage. The TMR10 V11.3 calculation algorithm was used and the skull was determined using the CT dataset. The 50% prescription line of 9 Gy and the 12 Gy dose to brain minus GTV was recorded. Mean dose to brain tissue minus GTV was recorded. Paired sample *t*‐tests were performed on Excel V16.77.1.

### 2.2 Sequential lesion addition to patient plans

The three BM patients were re‐established without any lesions, and lesions were introduced back based on size, largest first to smallest. Plans were generated in each of the systems (GammaPlan or Eclipse) for 1 target, then 2 targets, then adding 2 targets sequentially until the total original number of targets was reached. Each plan was normalized such that at least 99.5% of all GTVs were covered by 18 Gy. The plans were exported to MIM and the mean dose to brain (brain minus GTV) was recorded as a function of number of lesions introduced.

### 2.3 Separation of 50% isodose volumes

We sought to investigate the ability of the two systems to separate nearby targets. Therefore, the distance required for separation of 50% prescription dose volumes between two adjacent lesions was evaluated for the GK compared with microMLC and MLC based linac. Lesions of diameters 0.5 cm, 1.0 cm, and 1.5 cm were placed on an anonymized normal brain CT scan. One lesion was placed near the brain center. A second was varied in center‐to‐center distance from the central lesion in each of the superior/inferior (SI), right/left (RL), or anterior/posterior (AP) directions. Treatment plans giving doses of 18 Gy to at least 99.5% of each lesion were created in Eclipse. In GammaPlan, an appropriate shot was found to best conform to that size target—specifically 4 mm at an isodose prescription of 60% was used for a 0.5 cm target, with target coverage being at least 99.5%. For 1.0 cm targets, a shot size of 8 mm was used with an isodose prescription of 50%. Lastly for 1.5 cm targets, a shot size of 16 mm was used, with an isodose prescription of 65%. The edge‐to‐edge distance between the lesions was increased until the 9 Gy isodose lines (50% of the prescription dose) were separated between the two adjacent lesions.

### 2.4 Simulated plans with a matrix of lesions

Two structured arrangements of 27 brain lesions configured in an equally spaced cubic matrix were created, one with 0.5 cm targets, the other with 1.0 cm targets. The 1.5 cm targets were left out of the matrix simulation due to space limitations that prevented the inclusion of 27 targets. Each lesion was spaced 3.3 cm apart from one another along the principal axes. The resulting cube was 7.7 cm in length, width, and height. This cube size was the largest size that would comfortably fit inside the cranium on an example human brain CT. Treatment plans were made on an interval basis where three lesions were added per iteration, each with random locations on the grid. Dose was exported to MIM for calculation. Mean dose to brain was recorded.

## 3 RESULTS

**FIGURE 1:**
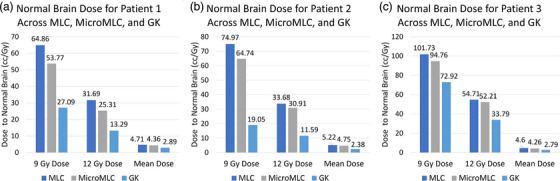
Plan comparisons among three planning techniques for (a) patient one, (b) patient two, and (c) patient three. Volume receiving 9 Gy Dose (V9 Gy) and 12 Gy Dose (V12 Gy) are in units of cc. Mean Dose received to whole brain is in units of Gy.

Patient plans were compared on GK and linac SRS regarding brain tissue dose as follows (Figure 1). For Patient 1, the GK system delivered 12 Gy to 13.29 cc of normal brain, which was significantly less than the 25.31 cc and 31.69 cc treated by the microMLC and MLC‐based Linac systems, respectively. Similarly, the mean dose was 2.89 cc of brain tissue with the GK system, compared to 4.36 cc with microMLC and 4.71 cc with MLC. This trend continued across patients. For Patient 2, the GK system again delivered 12 Gy to only 11.59 cc of normal brain, in contrast to 30.91 cc with microMLC and 33.68 cc with MLC. The mean dose for GK was 2.38 cc, compared to 4.75 cc for microMLC and 5.22 cc for MLC. Patient 3 received 12 Gy to 33.79 cc of brain with the GK system, which was less than the 52.21 cc and 54.71 cc treated by microMLC and MLC, respectively. The mean doses were 2.79 cc for GK, 4.26 cc for microMLC, and 4.6 cc for MLC). In comparison with microMLC, a paired sample t‐test comparing mean dose with GK gave a *p* value of 0.027. In a paired sample t‐test comparing mean dose between GK and MLC, a *p* value was calculated to be 0.024.

**FIGURE 2:**
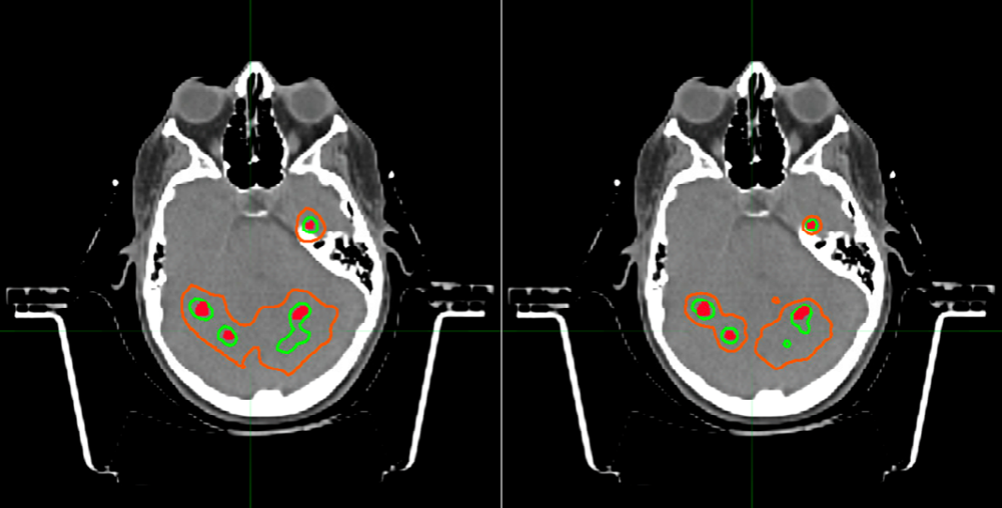
Dose cloud comparison for patient 3. Plans were exported to MIM and displayed with normalization for direct comparison. Left image is microMLC, and right image is GK. Lines in green represent the region receiving 18 Gy. Lines in orange represents region receiving 9 Gy. Solid red areas represent GTVs.

Figure 2 shows for patient 3 an example of two observed effects comparing microMLC based and GK systems. First, the microMLC plans do not as effectively separate the dose clouds of nearby targets. Second, microMLC plans are not as conformal for tiny isolated metastases. This led to further investigation.

To investigate the ability of the MLCs to separate multiple lesions, we generated plans adding the first two targets then every other target to target 30 (Patients 1–2) or target 18 (Patient 3). Initially, the differences in the doses across the systems were minimal for 1–2 targets. In Patient 1, treating the first lesion resulted in a mean dose of approximately 0.3 Gy to normal brain tissue across all treatment planning systems (TPS). However, as more lesions were incorporated, significant divergence in dose distributions became apparent. At 30 lesions, the mean dose escalated to 2.74 Gy using the GK system, compared to 4.76 Gy and 4.79 Gy with microMLC and MLC, respectively. For Patient 2, the initial addition of one lesion led to a mean dose to 0.2 cc of brain tissue. This increased significantly by target 30, where the GK system delivered a mean dose of 2.26 Gy, while the doses with microMLC and MLC reached 4.32 Gy and 4.35 Gy, respectively. Similarly, in Patient 3, the treatment of the first lesion delivered a mean dose of 0.3 Gy to brain tissue with GK, and slightly higher at 0.4 Gy with both microMLC and MLC systems. Upon incorporating up to the 18th lesion, the mean doses were 2.79 Gy for GK, 4.26 Gy for microMLC, and 4.6 Gy for MLC.

**FIGURE 3:**
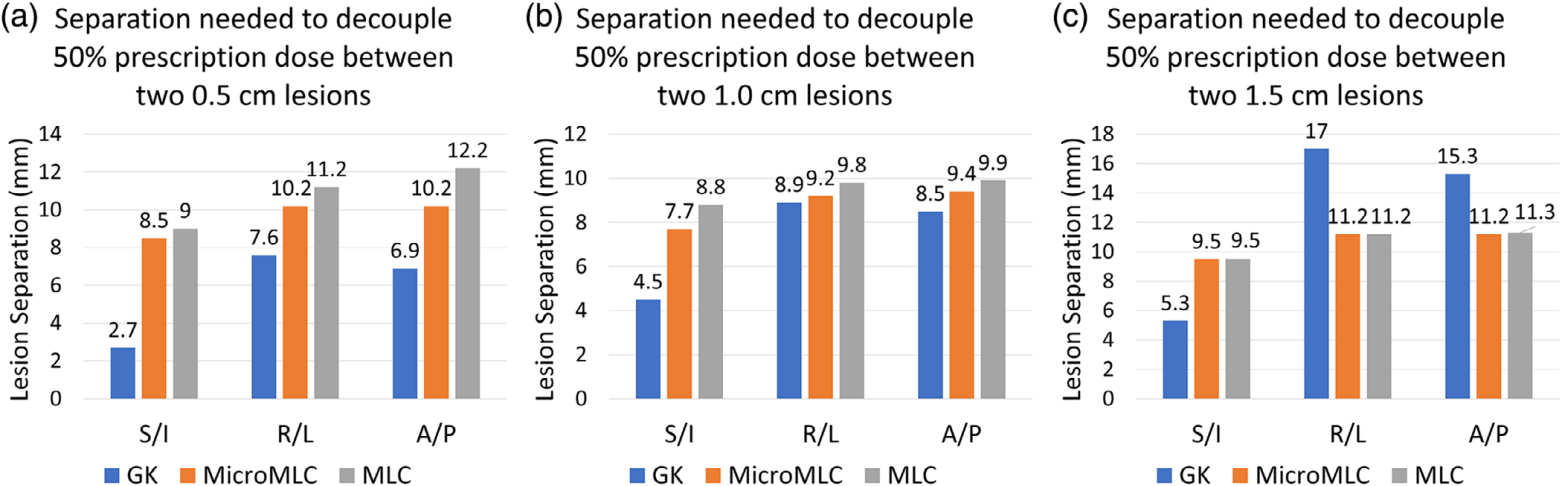
Comparison of distances separating 50% prescription dose volumes between two lesions dependent on system, considering lesion orientation. Lesion sizes: 0.5 cm (a), 1 cm (b), 1.5 cm (c). GK collimator sizes: 4 mm, 8 mm, 16 mm for 0.5 cm, 1 cm, and 1.5 cm lesions, prescribed to isodose lines of 60%, 50%, and 65%, respectively.

The distance required for separation of 50% prescription dose volumes between two adjacent lesions was evaluated for the GK compared with microMLC and MLC based linac (Figure 3). Findings varied based upon lesion size, TPS, and orientation. Essentially, GK separated the dose best between adjacent targets in the superior‐inferior axis. For example, for 0.5 cm lesions it only took 2.7 mm separation to separate 50% of prescription isodose compared to right‐left (7.6 mm) and anterior‐posterior (6.9 mm) axes. GK plans separated the 50% prescription isodose cloud more readily in all axes for 0.5 cm lesions (e.g., 7.6 mm right‐left for GK vs. 10.2 mm for microMLC vs. 11.2 mm for MLC), while the differences were smaller for 1.0 cm lesions and mixed for 1.5 cm lesions.

**FIGURE 4:**
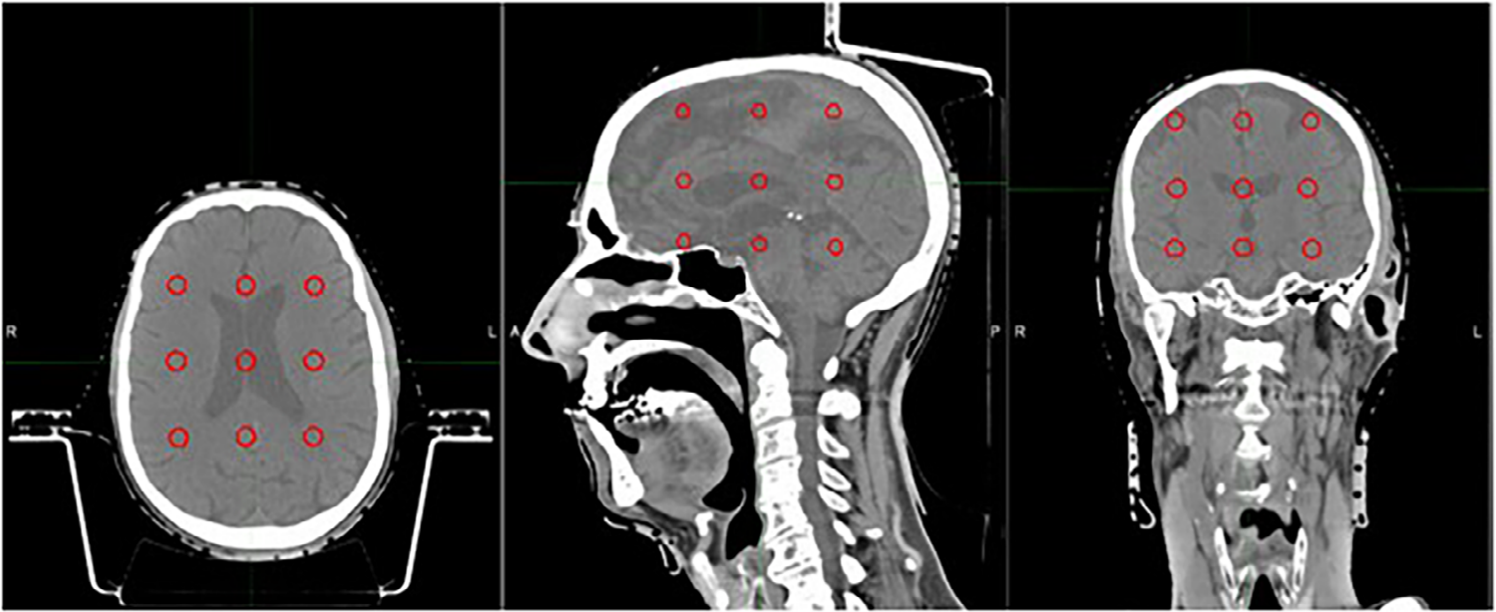
Matrix of 1.0 cm lesions seen in different orientations, 3.3 cm separated from each other in principal axes.

**FIGURE 5:**
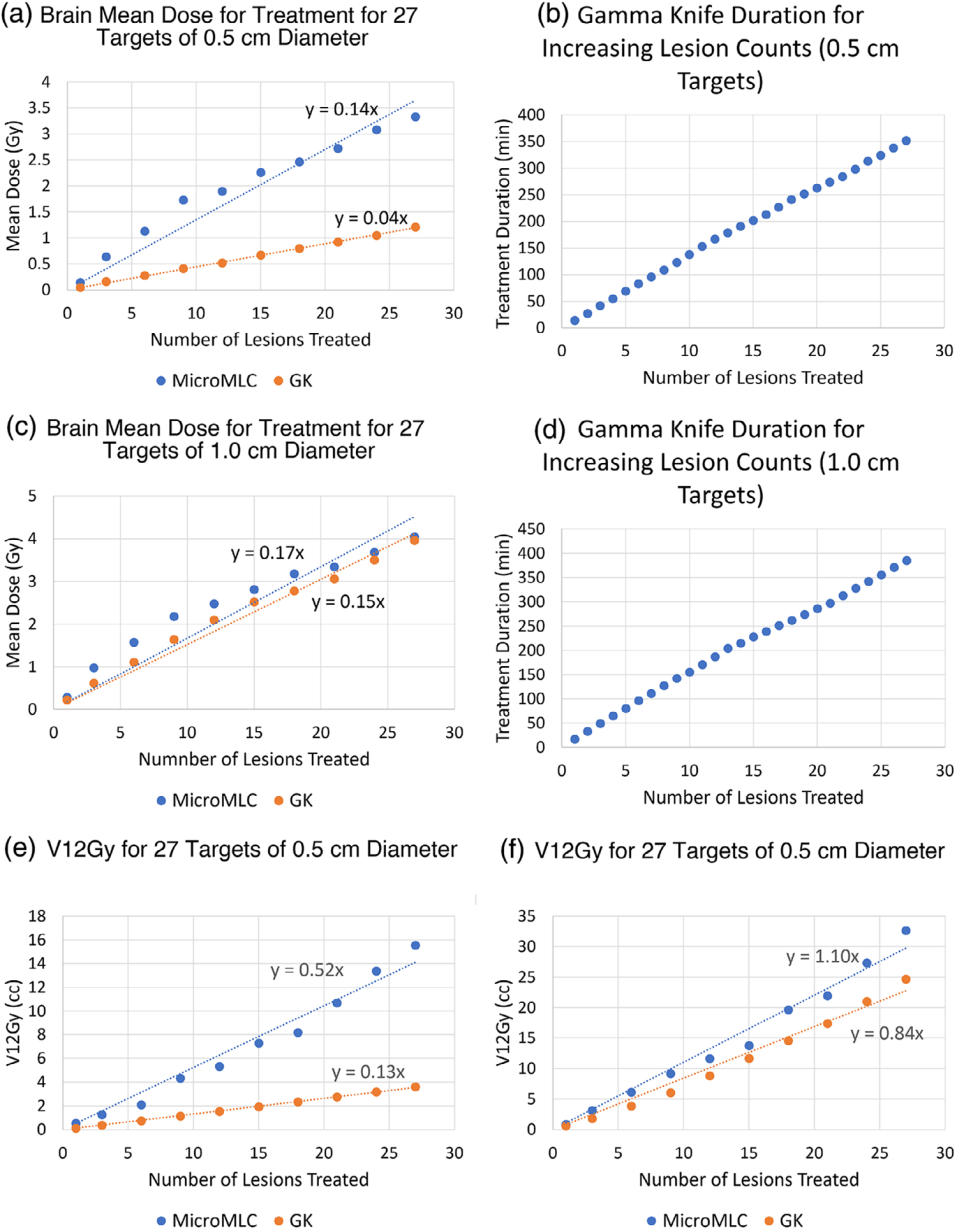
Dose accumulation and treatment metrics for adding lesions. (a) Dose accumulation for 0.5 cm lesions, (b) treatment time for 0.5 cm lesions, (c) dose accumulation for 1.0 cm lesions, (d) treatment time for 1.0 cm lesions, (e) V12 Gy for 0.5 cm lesions, and (f) V12 Gy for 1.0 cm lesions.

We created a matrix of lesions as described in the methods and shown in Figure 4. We hypothesized that there could be a limit of the ability of the MLCs to separate doses to high numbers of targets that would be reflected in the mean brain dose. However, no such limit was found, and the mean brain dose remained approximately linear as lesions were added (Figure 5).

Instead, we identified that for 0.5 cm lesions, there is a small difference in mean brain dose for the first lesion that becomes linearly additive for each lesion added. For example, microMLC shows increases from 0.14 Gy for the first lesion to 3.33 Gy for all 27 lesions. Conversely, the mean brain dose for the GK starts at 0.05 Gy and rises to 1.21 Gy. Linear regression identifies 0.14 Gy mean brain dose per 0.5 cm lesion on microMLC linac, compared to 0.04 Gy mean brain dose per 0.5 cm lesion on GK, or a difference of 0.1 Gy per lesion. For one or a small number of lesions, this is almost certainly trivial, however this balloons to a measured 2.12 Gy difference in mean brain dose between the two systems for all 27 lesions. When this study was repeated for lesions sized 1.0 cm, the differences are minimal, with microMLC giving a dose of 0.28 Gy for the first lesion to 4.04 Gy for all 27 lesions, and GK system starting at 0.22 Gy and ending at 3.96 Gy. Comparing the linear regressions, the difference in mean brain per target added between microMLC linac and GK was 0.02 Gy (0.17 Gy per target with microMLC, 0.15 Gy per target with GK).

Similar trends were observed when V12 Gy of normal brain was compared. For lesions measuring 0.5 cm, the microMLC system starts with V12 Gy of 0.53 cc, increasing sharply to 15.55 cc by the 27th lesion (linear regression V12Gy = 0.52 cc/lesion). In contrast, the GK system is a bit more gradual, starting at 0.13 cc for the first lesion and increasing to 3.6 cc by the 27th lesion (linear regression V12 = 0.13 cc/lesion). For 1.0 cm lesions, the difference was smaller but still present. The microMLC linac started at V12 Gy of 0.84 cc for the first lesion and escalating to 32.66 cc by the 27th lesion (linear regression V12Gy = 1.1 cc/lesion). The GK system, starting at 0.6 cc, increases to 24.7 cc by the 27th lesion (linear regression V12Gy = 0.84 cc/lesion).

## 4 DISCUSSION

Radiosurgery has evolved from an adjunct to whole‐brain radiation for few lesions to an independent technique for many lesions, now studied for nearly unlimited lesions.^16^, ^21^, ^22^ The NCIC CE.7 trial (NCT03550391), compared whole‐brain radiation to SRS for 5–15 brain metastases and now focuses on hippocampal avoidance whole‐brain radiation versus SRS for 5+ metastases. Improved systemic therapies have extended survival, raising questions about SRS as an alternative to repeat whole‐brain radiation. Our center, with both GK and Linac SRS, has addressed practical treatment challenges for these patients.

We planned three patients in this situation of a high number of brain metastases for SRS. One abstract has investigated this previously for as many as 53 brain metastases with similar results which also demonstrated improved brain dosimetry for GK compared to Linac SRS.^23^ Our goal here was to reproduce this in our patients and then identify the reasons for these dosimetric differences.

**FIGURE 6:**
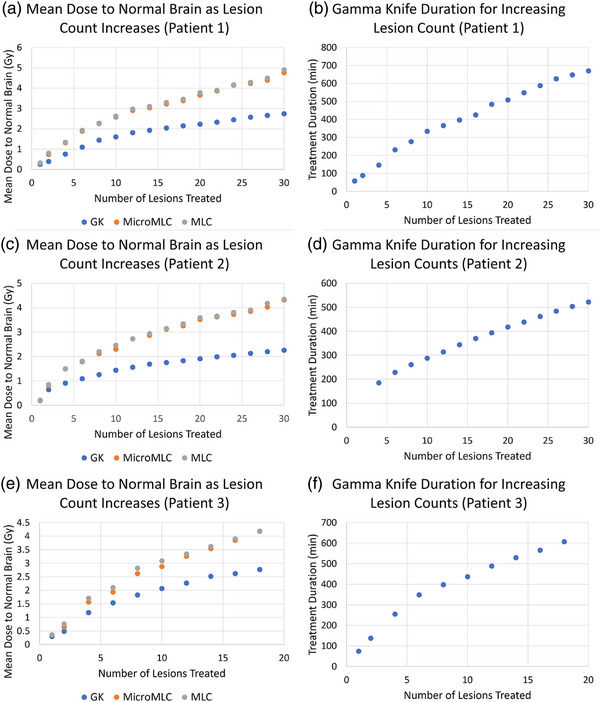
(a) Dose accumulation and GK treatment time for the addition of lesions for (a, b) patient one, (c, d) patient two, and (e, f) patient three.

There were several key findings. First, GK provides superior plans compared with MLC plans for high number brain metastases patients at the expense of excessive treatment times (Figure 1). For single targets this difference is quite small (on the order of 0.1 Gy of mean brain dose between GK and Linac SRS), but when treating many targets this multiplies relatively linearly to several Gy differences (Figures 3 and 6a). This may be a result of the sharper beam penumbra in Gamma Knife compared to linac. Differences in doses can also be attributed to variations in lesion size and distribution. For adjacent lesions, GK is better at separating dose clouds occurring over the interspacing brain, which contributes to its improved plan quality (Figure 3). This may again be partially explained by beam penumbral differences, but the dosimetric findings for linac are multi‐factorial since the physical beam characteristics are also shaped by how the MLC compensates for the penumbra using VMAT.^24^ A minor consideration to normal brain dose may also be interleaf leakage or MLC transmission, which was measured on our systems at 0.7% for the microMLC (6MV‐FFF) and 1.24% for the MLC (6MV‐FFF). Finally, the MLC's ability to separate dose clouds between distant lesions is equivalent to that of GK for larger targets, as demonstrated by the small difference in mean brain dose for 1.0 cm lesion cases (Figure 6c). It is important to note that HyperArc optimization is largely automated, while GK planning involves a more manual, skill dependent process. Despite this potential disadvantage for GK treatment plans, our results show that GK consistently achieved better dose sparing characteristics. This suggests that even with this disadvantage, GK is capable of potentially outperforming HyperArc optimization.

One limitation is that the clinical relevance of such dosimetric differences is unclear. There are several considerations. First, there is a common perception that SRS to many targets creates mean brain doses so high that it is equivalent to whole brain radiotherapy doses (e.g., 8 Gy in 1 fraction). Our mean brain values in actual patients were in the 2–5 Gy range despite the technique used, which is equivalent to a fraction of fractionated whole brain radiation (3 Gy x 10 fractions or 4 Gy x 5 fractions) and cannot be expected to have the same side effects as a full course of whole brain radiation. Previous studies have demonstrated that mean brain dose increases linearly with the number of lesions as well as total GTV volume, reinforcing the feasibility of treating numerous metastases within safe dosimetric limits. Additionally, findings highlight the importance of accounting for lesion size and volume when evaluating dosimetric parameters.^25^ Second, the radiation plans for GK are difficult to standardize across centers because there is significant variability in plan quality and source strength of Cobalt‐60. We used GK plans that created high conformality at the expense of time, but an interested party could possibly create faster plans.^26^, ^27^ Careful attention must be paid, as fast but non‐selective planning with large shots will likely negate part or all of the plan quality benefits of GK. The use of planning tumor volume (PTV) expansion is also controversial. While PTV has not been used historically for GK, frame‐based and mask‐based GK SRS is not without potential error.^28^, ^29^, ^30^ Questions remain involving the accuracy of single isocenter HyperArc and if it is adequate for treating lesions without PTV. Some groups treat without PTV, but the physical accuracy of mask‐based single isocenter linac based SRS treatments have been called into question.^31^, ^32^ For a direct comparison, no PTV was used, and the addition of PTV to either technique will worsen plan metrics.

In this study, we recognize that the number of patients included was small, which may limit the generalizability of the results. While differences in brain metastases configurations across patients may result in some variability, our findings closely align with results from idealized situations, suggesting robustness in overall trends.

One complication when comparing plans for many brain metastases in a single treatment is how to calculate or use conventional dose metrics such as conformity index and gradient index that are typically used for single lesions. The problem is that when treating closely spaced metastases, dose grids will include dose from adjacent metastases. Further, it is unclear how to handle dose clouds that do not separate between lesions. As such, we chose to look at whole brain metrics like mean brain and whole brain V12 Gy when comparing plans in this study. In theory, one could consider a whole brain conformity index as the prescription dose volume within the whole brain over the cumulative target volume or a similar whole brain gradient index as the volume receiving 50% of the prescription dose within the whole brain over the prescription dose within the whole brain.

We have come across a real‐world application where the NCIC CE.7 trial protocol limits V12 Gy to 30 cc over the brain. The protocol requires lowering of target prescription doses until V12Gy < 30 cc is met. Even at 18 Gy per target (CE.7 specifies 22 Gy ± 2 Gy for small lesions), doses would have to be reduced for patient 1 (MLC only), patient 2 (MLC and microMLC), and patient 3 (MLC and microMLC) while GK doses would only have to be reduced minimally to meet the V12Gy < 30cc threshold for patient 3 and could likely be increased to protocol dose on GK for patients 1 and 2. Complying with that treatment protocol using HyperArc will lead to decreased target dose which would likely reduce target control.^33^ Future work from CE.7 and others should provide neurocognitive outcomes that may suggest whether these differences in V9 Gy, V12 Gy, and mean brain dose observed here have any clinical relevance.

## 5 CONCLUSION

Gamma Knife Perfexion, compared to single isocenter MLC‐based linac (HyperArc), provides superior plans for patients with adjacent and multiple small brain metastases. This is a result of the inferior capabilities of single isocenter MLC (even microMLCs) delivery to separate dose clouds from adjacent lesions or provide highest conformity to small brain metastases (5 mm). However, GK treatment times are excessively long (as much as 10+ h) for 18‐33 brain metastases. The clinical relevance of these dosimetric differences are unclear. Clinical trials allowing for both GK and single isocenter linac treatments should be aware of significant dosimetry differences between the two systems that could result in lower doses for Linac SRS per protocol that could confound results.

## AUTHOR CONTRIBUTIONS

Dr. Abdou acquired and analyzed data and wrote the manuscript. Dr. Almeida, Bossart, and Monterroso contributed to data acquisition, analysis, and writing. Dr. Mellon conceived of the project and contributed to data analysis and writing.

## CONFLICT OF INTEREST STATEMENT

The authors declare that they have no known competing financial interests or personal relationships that could have appeared to influence the work reported in this paper.

## Supporting information



Supporting Information

Supporting Information

Supporting Information
