# Supplementary figures and images for: Stereotactic radiosurgery for multiple small brain metastases using gamma knife versus single‐isocenter VMAT: Normal brain dose based on lesion number and size
Source: J Appl Clin Med Phys. 2025 Mar 19;26(6):e70065. doi: 10.1002/acm2.70065 (PMC12148777; doi:10.1002/acm2.70065)

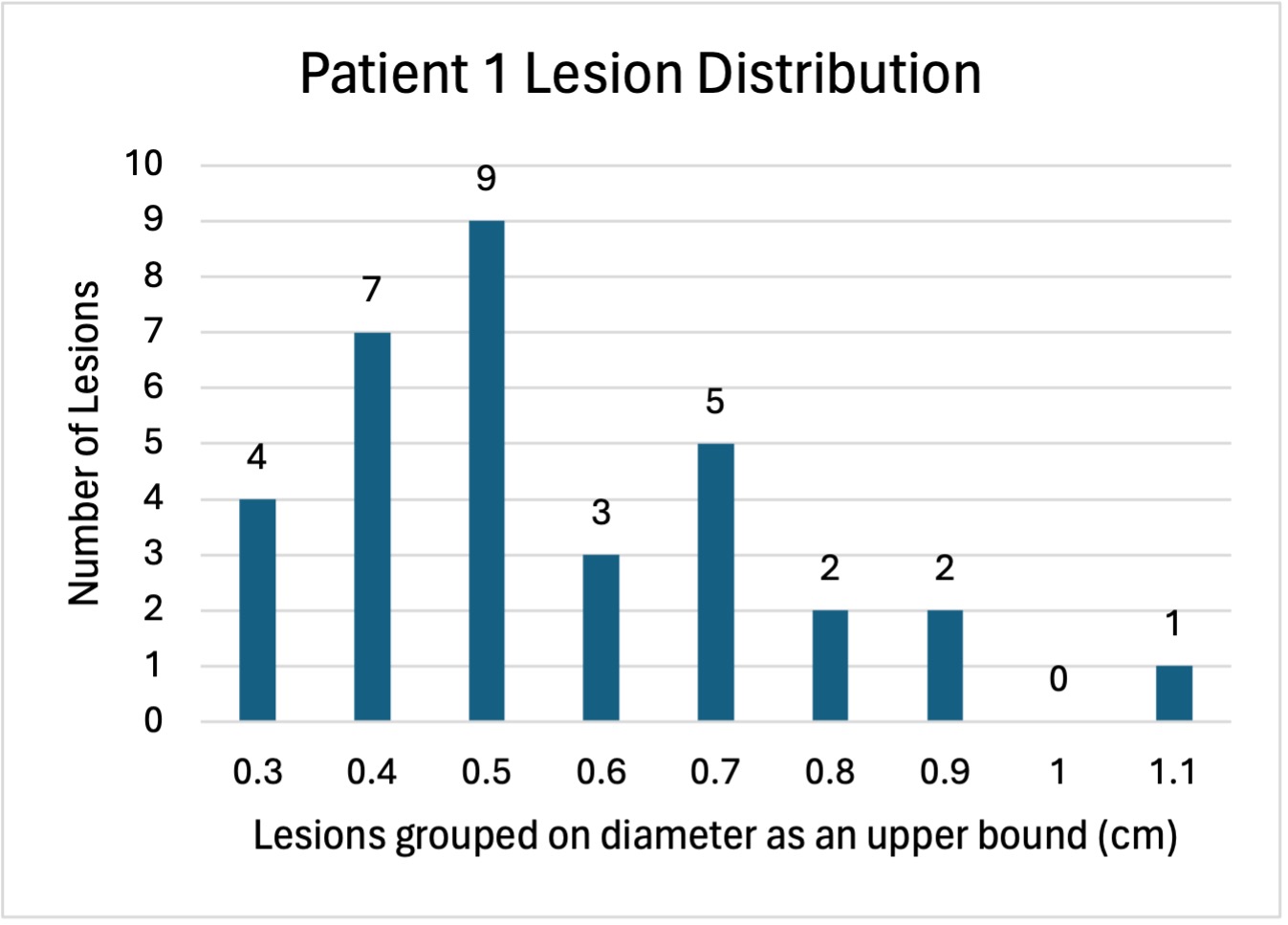

Supplement: Supplementary file 1 — Supporting Information [file ACM2-26-e70065-s001.jpg]

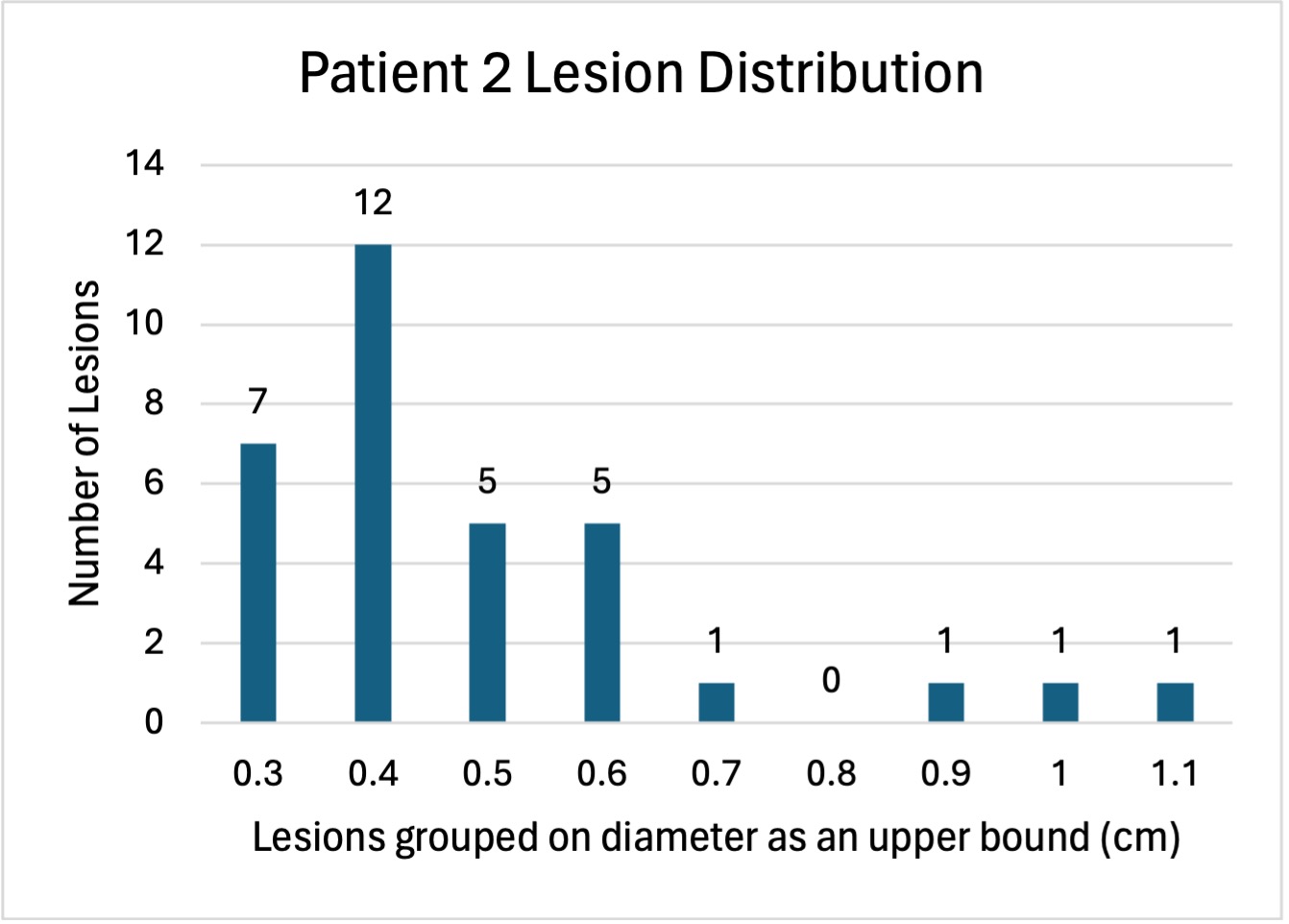

Supplement: Supplementary file 2 — Supporting Information [file ACM2-26-e70065-s002.jpg]

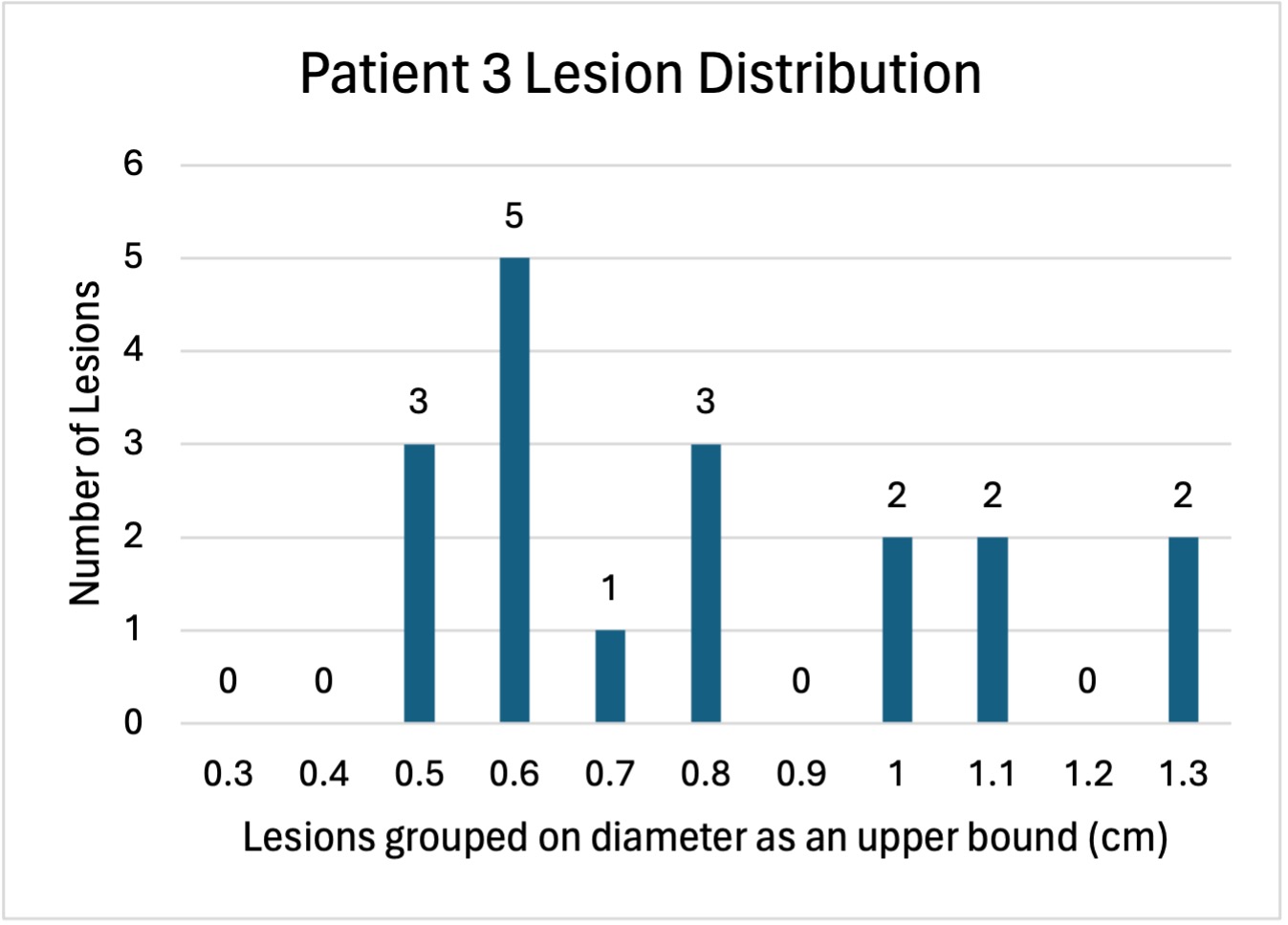

Supplement: Supplementary file 3 — Supporting Information [file ACM2-26-e70065-s003.jpg]
